# Blarcamesine for the treatment of Early Alzheimer's Disease: Results from the ANAVEX2-73-AD-004 Phase IIB/III trial

**DOI:** 10.1016/j.tjpad.2024.100016

**Published:** 2025-01-01

**Authors:** Stephen Macfarlane, Timo Grimmer, Ken Teo, Terence J O'Brien, Michael Woodward, Jennifer Grunfeld, Alastair Mander, Amy Brodtmann, Bruce J. Brew, Philip Morris, Cathy Short, Susan Kurrle, Rosalyn Lai, Sneha Bharadwaj, Peter Drysdale, Jonathan Sturm, Simon J.G. Lewis, David Barton, Chris Kalafatis, Saif Sharif, Richard Perry, Nicholas Mannering, J．Emer MacSweeney, Stephen Pearson, Craig Evans, Vivek Krishna, Alex Thompson, Malathy Munisamy, Neel Bhatt, Aliya Asher, Sandra Connell, Jennifer Lynch, Sterre Malou Rutgers, Paul LJ Dautzenberg, Niels Prins, Patrick Oschmann, Lutz Frölich, Pawel Tacik, Oliver Peters, Jens Wiltfang, Alexandre Henri-Bhargava, Eric Smith, Stephen Pasternak, Andrew Frank, Howard Chertkow, Jennifer Ingram, Ging-Yuek Robin Hsiung, Rodney Brittain, Carmela Tartaglia, Sharon Cohen, Luca M Villa, Elizabeth Gordon, Thomas Jubault, Nicolas Guizard, Amanda Tucker, Walter E Kaufmann, Kun Jin, William R Chezem, Christopher U Missling, Marwan N Sabbagh

**Affiliations:** a The Dementia Centre, HammondCare, Melbourne, Victoria, Australia; b Technical University of Munich, School of Medicine and Health, Klinikum rechts der Isar, Munich, Germany; c The Department of Neuroscience, The School of Translational Medicine, Alfred Hospital, Monash University, Melbourne, Victoria, Australia; d University of Melbourne and Austin Health, Ivanhoe, Victoria, Australia; e Peninsula Therapeutic & Research Group Pty Ltd, Frankston, Victoria, Australia; f Geelong Private Medical Centre, Geelong, Victoria, Australia; g Royal Melbourne (RMH) Parkville, Victoria (Australia) and Eastern Clinical Research Unit, Monash University, Box Hill, Victoria, Australia; h St Vincent's Hospital Sydney, Darlinghurst, New South Wales, Australia; i Gold Coast Memory Disorders Clinic, Southport, Queensland, Australia; j The Queen Elizabeth Hospital, Woodville South, South Australia, Australia; k Hornsby Ku-ring-gai Hospital, Hornsby, New South Wales, Australia; l KaRa MINDS, Macquarie Park, New South Wales, Australia; m Australian Alzheimer's Research Organization, Nedlands, Western Australia, Australia; n Delmont Private Hospital, Glen Iris, Victoria, Australia; o Central Coast Neurosciences Research, Tumbi Umbi, New South Wales, Australia; p Brain and Mind Centre, University of Sydney, Camperdown, New South Wales, Australia; q NeuroCentrix, Noble Park, Victoria, Australia; r King's College London, London, United Kingdom; s Southern Health NHS Foundation Trust Memory Assessment & Research Centre, Southampton, United Kingdom; t Imperial College Healthcare NHS Trust, London, United Kingdom; u Re:Cognition Health, Guildford, United Kingdom; v Re:Cognition Health, London, United Kingdom; w Re:Cognition Health, Plymouth, United Kingdom; x MAC Clinical Research, Barnsley, United Kingdom; y MAC Clinical Research, Blackpool, United Kingdom; z MAC Clinical Research, Cannock, United Kingdom; aa MAC Clinical Research, Leeds, United Kingdom; bb MAC Clinical Research, Liverpool, United Kingdom; cc MAC Clinical Research, Manchester, United Kingdom; dd MAC Clinical Research, Stockton-on-Tees, United Kingdom; ee Glasgow Memory Clinic, Motherwell, United Kingdom; ff Brain Research Center, Amsterdam, the Netherlands; gg Brain Research Center, Den Bosch, the Netherlands; hh Brain Research Centre, Zwolle, the Netherlands; ii Klinikum Bayreuth, Bayreuth, Germany; jj Central Institute of Mental Health (CIMH), Mannheim, Germany; kk University Hospital Bonn, Bonn, Germany; ll Charité University Medicine, Berlin, Germany; mm Clinic for Psychiatry and Psychotherapy, Göttingen, Germany; nn Vancouver Island Health Authority, Victoria, British Columbia, Canada; oo Healthy Brain Aging Laboratories, University of Calgary, Calgary, Alberta, Canada; pp Parkwood Institute/Western University, London, Ontario, Canada; qq Bruyere Continuing Care, Ottawa, Ontario, Canada; rr BayCrest Health Sciences, Toronto, Ontario, Canada; ss Kawartha Centre - Healthy Aging Redefined, Peterborough, Ontario, Canada; tt University of British Columbia Hospital, Vancouver, British Columbia, Canada; uu True North Clinical Research, Halifax, Nova Scotia, Canada; vv Toronto Western Hospital, Toronto, Ontario, Canada; ww Toronto Memory Program, Toronto, Ontario, Canada; xx QYNAPSE SAS, Paris, France; yy QYNAPSE Canada Inc. Montréal, Canada; zz Anavex Life Sciences, New York, NY, USA; ab Barrow Neurological Institute, St. Joseph's Hospital and Medical Center, Phoenix, Arizona, USA

**Keywords:** Blarcamesine, Autophagy, Sigma-1 receptor, Randomized clinical trial

## Abstract

**Background:**

There are no approved oral disease-modifying treatments for Alzheimer's disease (AD).

**Objectives:**

The objective of this study was to assess efficacy and safety of blarcamesine (ANAVEX®2-73), an orally available small-molecule activator of the sigma-1 receptor (SIGMAR1) in early AD through restoration of cellular homeostasis including autophagy enhancement.

**Design:**

ANAVEX2-73-AD-004 was a randomized, double-blind, placebo-controlled, 48-week Phase IIb/III trial.

**Setting:**

Multicenter - 52 medical research centers/hospitals in 5 countries.

**Intervention:**

508 participants with early AD (Stage 3) were randomized to receive either blarcamesine (*n* = 338) in medium dose group 30 mg or in high dose group 50 mg or placebo (*n* = 170) oral capsules once daily for 48 weeks. Participants in these groups were offered to enroll into the open-label-extension study ATTENTION-AD, which completed June 2024, ClinicalTrials.gov Identifier NCT04314934.

**Measurements:**

The co-primary cognitive and functional outcomes were assessed as change in ADAS-Cog13 and ADCS-ADL from baseline to 48 weeks. The outcomes include the secondary outcome CDR-SB and biomarkers from the A/T/N spectrum, plasma Aβ42/40-ratio and global brain volume changes measured by MRI. All clinical endpoints were analyzed using mixed model for repeated measures (MMRM), plasma biomarker measurements were analyzed by Welch's *t*-test, and volumetric MRI scans were analyzed by general linear model.

**Results:**

Among 462 randomized participants in the intent-to-treat population (mean age, 73.7 years; 225 [48.7%] women), 338 (73.2%) completed the trial. The co-primary outcome was met under the multiplicity control rule, since the differences in the least-squares mean (LSM) change from baseline to 48 weeks between the prespecified blarcamesine and placebo groups for ADAS-Cog13 was significant at a level of *P* < 0.025 and for CDR-SB was significant at a level of *P* < 0.025, while ADCS-ADL did not reach significance at Week 48 (ADAS-Cog13 difference of -2.027 [95% CI -3.522 to -0.533]; *P* = 0.008; CDR-SB difference of -0.483 [95% CI -0.853 to -0.114]; *P* = 0.010; ADCS-ADL difference of 0.775 [95%CI -0.874 to 2.423]; *P* = 0.357). Plasma Aβ42/40-ratio increased significantly with blarcamesine group vs. placebo, (*P* = 0.048) and whole brain volume loss was significantly decreased (*P* = 0.002). Participants in the full safety population with ≥1 serious treatment-emergent adverse events (TEAEs) occurred in 56 participants (16.7%) in the blarcamesine and 17 (10.1%) in the placebo group. Common TEAEs included dizziness, which was transient and mostly mild to moderate in severity. One death in the blarcamesine group and 1 in the placebo group were both not considered treatment related.

**Conclusions:**

Blarcamesine, demonstrating a safety profile with no associated neuroimaging adverse events, significantly slowed clinical progression by 36.3% at 48 weeks with blarcamesine group as well as the individual 30 mg (by 34.6%) and 50 mg (by 38.5%) blarcamesine groups vs. placebo on the prespecified primary cognitive endpoint ADAS-Cog13. The prespecified secondary endpoint CDR-SB, which is used as the sole primary endpoint in recent successful AD drug submissions, is significantly improved at Week 48 with blarcamesine relative to placebo. The findings are supported by biomarkers from the A/T/N spectrum, including plasma Aβ42/40-ratio and reduction of whole brain atrophy. Additionally, the prespecified *SIGMAR1* gene variant subgroup analysis confirmed beneficial clinical effect of blarcamesine group through upstream SIGMAR1 activation - subjects with the common SIGMAR1 wild-type gene (excluding carriers of the mutated SIGMAR1 rs1800866 variant) experienced an even greater significant clinical benefit with slowed clinical progression by 49.8% at 48 weeks on the prespecified primary cognitive endpoint ADAS-Cog13. Oral once daily blarcamesine could represent a novel treatment in early AD and be complementary or alternative to anti-beta amyloid drugs.

## Introduction

By 2050, 1 in 85 people worldwide will be diagnosed with Alzheimer's disease (AD) [1]. At current estimates, approximately 60 million persons are living with dementia worldwide [2], and this represents a huge healthcare burden on patients, families and health systems worldwide. AD constitutes an estimated 60–80% of all dementias [3]. In the United States alone, health care and long-term care for people with AD and other dementias are projected to reach $1 trillion by 2050 (in 2023 dollars) [3].

The clinical and pathological presentation of AD is highly heterogeneous [4], being influenced by interactions between genotype, environment, cognitive reserve, and a range of demographic factors, among other determinants. Besides β-amyloid and tau, which capture only a portion of the biological mechanisms underlying AD, there is a growing appreciation for the co-occurrence of other concurrent pathologic insults, and an understanding that a more comprehensive or upstream approach is necessary to address the heterogeneous pathologies underlying AD. Restoring cellular homeostasis through activation of an upstream, endogenous pathway for clearing protein aggregates, including autophagy enhancement might be a promising approach with the potential for broad application. It would also avoid the risk of serious complications such as Amyloid Related imaging Abnormalities (ARIA) which can be life-threatening [5], [6], [7].

The overall mixed success of amyloid-targeting treatments [8], [9], [10], [11] and their potential for severe adverse events (AEs) [12], [13] has highlighted the need for safer effective treatments. Complex logistical procedures and associated high costs of treatment mean there is still an unmet need for scalable, orally bioavailable lines of treatment. SIGMAR1 receptors are abundantly expressed in the brain [14] and SIGMAR1 agonists such as blarcamesine have demonstrated effects in slowing neurodegenerative diseases [15], [16], [17]. Therapies that safely reduce neurodegeneration in AD could be complementary or alternative to existing treatments.

Blarcamesine (ANAVEX®2–73) is an oral drug candidate that restores cellular homeostasis by targeting SIGMAR1 and muscarinic receptors. Binding of SIGMAR1 agonists in the central nervous system (CNS) alters oligomeric forms of SIGMAR1 facilitating interaction with numerous client proteins to cause effect [14], [18], [19]. Blarcamesine has demonstrated *in-vivo* ability to improve elderly immune systems by making cells more able to clear out their waste, in a process called autophagy enhancement [20], and SIGMAR1 activation drives pro-survival pathways including mitochondrial function [21], lipid metabolism [22], and the endoplasmic reticulum stress response [15], all known to be relevant in the pathophysiology of neurodegenerative diseases. The neuroprotective cascade from SIGMAR1 activation may also reduce chronic disease related neuroinflammation [16] and provide an innate resistance to neurodegeneration [17].

Our Phase IIb/III trial in early AD sought to further our understanding on the safety and efficacy of blarcamesine in slowing disease progression and reducing neurodegeneration in patients with Alzheimer's disease. The trial hypothesis was that blarcamesine would have beneficial effects on outcomes in the treatment of early AD. We report here key findings from primary and secondary clinical and biomarker outcomes.

## Methods

### Study design

The ANAVEX2-73-AD-004 trial was a Phase IIb/III 48-week randomized, double-blind placebo-controlled, multicenter, international trial of blarcamesine in early AD. After completion of the placebo-controlled 48-week study, participants were offered to enroll into a 96-week open label extension (OLE) study ATTENTION-AD (ClinicalTrials.gov Identifier NCT04314934), which completed in June 2024. The 48-week study was conducted at 52 sites across 5 countries; Australia (19 sites), United Kingdom (15 sites), Canada (10 sites), Germany (5 sites) and Netherlands (3 sites) which enrolled 508 participants between August 27, 2018, and June 28, 2022, with database lock on November 17, 2022 (ClinicalTrials.gov Identifier: NCT03790709) [23]. Ethics review committees and institutional review boards approved the study protocol at each study site. Written informed consent was obtained from study participants or legally authorized representatives prior to participating in the study. An independent data and safety monitoring board oversaw the safety of participants and reviewed safety data periodically throughout the study. The study was conducted in accordance with the Declaration of Helsinki, the International Conference on Harmonization Good Clinical Practice Guidelines, and local regulatory and ethics requirements.

### Participants

**Fig. 1:**
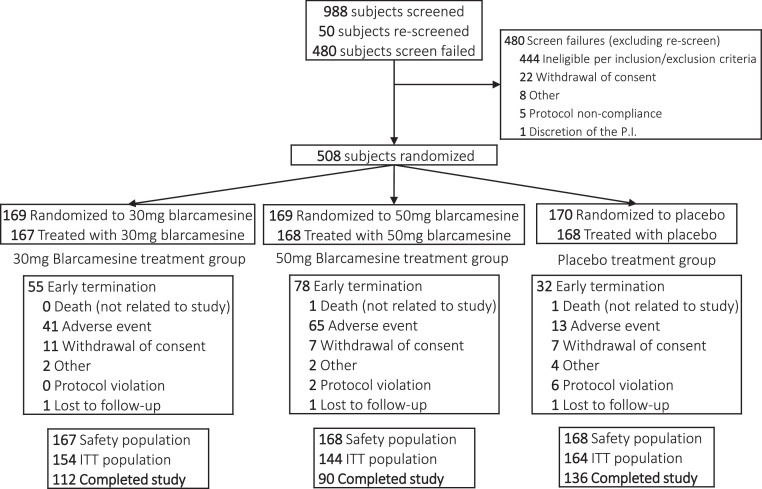
Flowchart of patient screening, enrollment, discontinuation, and completion.

Patients aged 60 to 85 years who met the National Institute on Aging (NIA) – Alzheimer's Association 2011 criteria for diagnosis of early-stage mild dementia due to AD or mild cognitive impairment due to AD [24], [25], [26] were eligible to participate in this study, with one of the following additional criteria required to support a diagnosis of AD: (a) historic or current record of CSF assessment compatible with AD, cut off values of amyloid beta (Aβ)42 < 1054 pg/mL, total Tau (tTau) >213 pg/mL, phosphorylated Tau (pTau) >21.3 pg/mL, and Aβ42/Aβ40 ratio <0.064 or CSF pTau181 >27 pg/mL (irrespective of the Aβ42/Aβ40 ratio) by automated Elecsys® CSF biomarkers assays (Roche Diagnostics) or comparable commercially used CSF assays, or (b) historic record of PET scan (amyloid scan or FDG-PET) within 36 months of screening, or (c) historic CT or MRI scan within 18 months of screening consistent with a diagnosis of Alzheimer's disease [24], [25], [26]. A Mini-Mental state examination (MMSE) score of 20 to 28 at the screening and randomization visits [27] and a Free and Cued Selective Reminding Test (FCSRT) recall score of ≤17 or total recall score <40 were also required [28], [29]. Patients on acetylcholinesterase inhibitors or other cognitive enhancing medications such as memantine, supplements, or nutraceuticals used to treat early AD were required to remain on stable doses for at least 90 days prior to screening. A complete flowchart of patient screening and enrollment is provided as Fig. 1.

Study outcome measures were obtained at baseline study entry and at weeks 12, 24, 36 and 48. MRI assessments and blood draws for plasma AD pathophysiologic biomarkers were obtained at baseline study entry and Week 48.

### Randomization and intervention

Randomization was performed by a third-party company using a proprietary validated and ISO certified program. The randomization code was generated on a remote server and randomized patients by assigning and shuffling blocks representing assigned treatment groups and randomization parameters, using a base block size of six. For each enrolled subject, at the time of enrollment, site staff entered the subject's information into the randomization server, which automatically randomized the subject and assigned an anonymized ID. Prior to unblinding, the data were only accessible to the third-party logistics team and selected members of the development team for programming purposes. All participants, care providers, investigators, data analysis team members, and other related personnel were blinded for the duration of the study.

Study participants were randomized to receive a daily oral dose of either placebo or blarcamesine at consistent timepoints relatively early in the morning with target dose of 30 mg or 50 mg daily for 48 weeks (ratio 1:1:1) in a flexible treatment titration design. At the start of the study participants underwent a 2-week treatment titration period which was modified in a protocol amendment to 3 weeks; over this period the treatment was up-titrated each week to the assigned target dose, maintaining blinding of treatment and dose. Irrespective of the target dose, the study protocol allowed for dose down titration, which was permitted for any reason, and similarly during the maintenance period when participants were required to maintain a minimum dose of 10 mg, resulting in the two active treatment groups receiving relatively similar treatment doses over the course of the study regardless of their initially assigned target dose. (Supplemental Table 1) Following the study rationale, the two active arms were analyzed separately and also combined to form a single active blarcamesine group and compared with placebo in the analysis. The single blarcamesine group vs placebo will be the primary analysis. The two separated arms vs placebo provide the support evidence.

### Outcomes

#### Clinical endpoints

The co-primary outcomes were reduction in cognitive decline assessed from baseline over 48 weeks with blarcamesine compared to placebo using the 13-item Alzheimer Disease Assessment Scale-Cognition (ADAS-Cog13), and reduction in decline of the ability to perform daily activities assessed from baseline over 48 weeks with blarcamesine compared to placebo using the Alzheimer's Disease Cooperative Study – Activities of Daily Living (ADCS-ADL) Scale [23].

The secondary outcome was the reduction in cognitive and functional decline assessed from baseline over 48 weeks with blarcamesine compared with placebo using the Clinical Dementia Rating Scale Sum of Boxes (CDR-SB) [23], [30], [31].

One exploratory clinical endpoint specified in the protocol was also analyzed: the questionnaire-based Clinical Global Impression – Improvement (CGI-I) scale.

#### Plasma Aβ42/Aβ40 ratio and plasma Nf-L, p-Tau (181), and p-Tau (231) biomarker endpoints

Analysis of biomarkers for available blood specimens was conducted using single molecule array immunoassay (SIMOA HD-X), enzyme-linked immunosorbent assay (ELISA), or enzyme-linked lectin assay (ELLA) technology depending on the analyte.

As exploratory biomarker efficacy endpoints, plasma levels of Aβ40 and Aβ42 were quantified at baseline and Week 48 by ELISA and used to calculate Aβ42/Aβ40 ratio, an established indicator of amyloid beta deposition in the brain. Plasma levels of neurofilament light chain (Nf-L), p-Tau (181), and p-Tau (231) were quantified by SIMOA HD-X at baseline and Week 48 as exploratory biomarker efficacy endpoints.

#### MRI biomarker endpoints

As an additional biomarker efficacy endpoint, structural MRI scans were performed at baseline and Week 48 and used to quantify changes in brain volume over the course of the study. Specifically, based on 3D T1-weighted images, volumes of whole brain, total white matter, total grey matter, and lateral ventricles were quantified and analyzed in terms of annualized percent change from baseline. Efficacy was evaluated as the reduction of brain volume decrease compared to placebo, while efficacy for lateral ventricles was evaluated as the reduction of brain cavities (fluid filled structures) volume increase compared to placebo.

#### SIGMAR1 gene variant genotyping [common SIGMAR1 gene (WT) and variant (rs1800866)]

As a prespecified exploratory endpoint of the study, clinical efficacy measurements were compared for subgroups based on absence or presence of a *SIGMAR1* gene variant (rs1800866 *T* > *G* missense variant) to assess the impact of this genetic variant on clinical efficacy. The common *SIGMAR1* allele for the rs1800866 variant is T, the WT = Wild Type (∼80%−70% of the general population), while ∼20%−30% of the general population carry the G allele, the mutated *SIGMAR1* gene variant [32].

### Sample size calculation

Sample size and power calculations were based on a simulation approach with several planned scenarios and assuming co-primary endpoints (ADAS-Cog13 and ADCS-ADL). The sample size calculation assumes the mean difference between either blarcamesine arm and placebo of 1.5 points (SD=4.5) in the ADAS-Cog and ADCS-ADL with at least 90% power using a two-sample *t*-test with alpha = 0.05 (2-sided). For the calculation of power concerning co-primary endpoints, conservatively assuming that power can be independently calculated [33], this will achieve at least 80% power for two endpoints. A 33% dropout rate was considered in estimating the sample size based on earlier studies. Therefore, 509 participants would need to be enrolled to allow for an anticipated 342 completers, i.e., 228 patients per combined treatment and 114 per placebo arm, respectively.

### Statistical analyses

Statistical analyses were done with SAS version 9.4 (SAS Institute) or R Project version 4.2.3 (R Foundation).

#### Analysis of clinical endpoints

The study protocol prespecified the reduction in decline assessed from baseline over 48 weeks with blarcamesine compared to placebo for the respective co-primary (ADAS-Cog13 and ADCS-ADL) and secondary (CSR-SB) endpoints using the mixed effects model. Hence, all prespecified clinical endpoints, including ADAS-Cog13, ADCS-ADL, CDR-SB, and CGI-I were analyzed using a linear mixed model (mixed model for repeated measures; MMRM). The MMRM analysis method is the convention used for regulatory filings and was used as the primary analysis method in all recent regulatory decisions for aducanumab [5] and lecanemab [7], as well as donanemab [6] with similar specifications.

Primary and secondary analyses were carried out in the protocol-specified analysis population, the “intent-to-treat” (ITT) population, which corresponds to what is typically termed “modified intent-to-treat” (mITT) and was defined as all randomized patients who received at least one study dose and had at least one post-dose clinical measurement.

The change of clinical scores from baseline to Week 48 was analyzed as the dependent variable, with treatment and visit week as fixed effects, treatment-by-visit as interaction effect, and baseline score, country, baseline concomitant AD medication, natural logarithm of the baseline plasma Nf-L concentration, baseline MMSE status, and SIGMAR1 receptor gene variant genotype status (single-nucleotide polymorphism SIGMAR1 rs1800866 presence or absence) as covariates in the model. For CGI-I, baseline CGI-S score was used as baseline. The primary comparison was the contrast (difference in the least squares mean) between blarcamesine and placebo at the last visit (Week 48), which was performed for the active treatment group as well as separately for the assigned (30 mg and 50 mg) treatment groups. For the primary analysis, the model assumed the missing data to be missing at random without imputation.

#### Analyses of plasma biomarkers

The plasma biomarker endpoints were assessed at the baseline and the end of the study (Week 48). Statistical significance was assessed with a *t*-test, using a significance threshold of *p* < 0.05 (*), *p* < 0.01 (**), or *p* < 0.001 (***). Considering the heterogeneity presented in these data sets, a Welch's unequal variance option was used.

#### Analysis of MRI biomarkers

All imaging processing was performed blind to participant group allocation (treatment or placebo). MRI data from baseline and final visit (48 weeks) were analyzed using QyScore® [34], [35] (www.qynapse.com/qyscore). QyScore® is intended for automatic labelling, visualization and volumetric quantification of brain structures and lesions from MR images.

3D T1-weighted images were processed through the QyScore® workflow [34]. Within this workflow, images were processed through Statistical Parametric Mapping software (SPM12) [36], which includes bias field correction to correct for inhomogeneities in the magnetic field, and segmentation into three tissue classes: grey matter, white matter, and cerebrospinal fluid. For each subject, overall volumes were produced and exported for regions including Whole Brain Volume (combined total grey matter and white matter volumes), Whole Brain White Matter, and Whole Brain Grey Matter. The Lateral Ventricles were segmented using an implementation of a 3D U-Net deep learning segmentation algorithm (called BGCVBS) [37].

The least-squares mean treatment difference of the annualized percent change MRI data was analyzed using a general linear model with adjustments for treatment group, baseline volume, and baseline MMSE status.

#### Safety objectives - adverse events

Safety objectives were evaluated by the incidence of AEs and serious AEs in the full safety population for both active and placebo groups and were summarized according to event frequency by treatment assignment.

#### Missing data

For the primary analysis, the MMRM model assumed the missing data was missing at random without imputation. The missing data for MMRM analyses were handled by the likelihood base mixed effect model and the efficacy parameters were estimated by incorporating all the observations.

#### Sensitivity analysis

The primary analyses assume that missing efficacy assessments are missing at random (MAR). To assess the robustness of the primary analyses, a tipping point analysis under missing not at random (MNAR) assumption was conducted for ADAS-Cog13. In this analysis, 100 datasets were first generated with assumptions of MAR using SAS PROC MI. The missing not at random was realized by worsening imputed values in the active arm with increment of 0.02. or by improving imputed values in placebo arm with increment of 0.04. The primary MMRM model was applied to each of the 100 worsening or improving datasets. With each incremental change, these results from imputed data were combined using Rubin's combination rules, with SAS PROC MIANALYZE. The process stops when the primary model result is no longer significant.

## Results

Of 988 participants screened, 508 were enrolled and randomized, and among 462 randomized participants in the ITT population (mean age, 73.7 years; 225 [48.7%] women), 338 (73.2%) completed the trial. 338 were assigned to receive blarcamesine and 170 were assigned to receive placebo (Fig. 1). Baseline characteristics of the ITT population are summarized by blarcamesine group (*n* = 298), assigned to 30 mg group (*n* = 154), assigned to 50 mg group (*n* = 144), and placebo (*n* = 164) group (Table 1). Due to the prespecified flexible dosing design of the study, the 30 mg and 50 mg assigned dosage arms reached quite similar average cumulative exposure at each study visit (Supplementary Table 1); hence the combined blarcamesine group vs placebo is the primary analysis and supported by the comparison of separated dose groups vs placebo. Study drug compliance (actual days of exposure/planned days of exposure) was high, with a mean of 96% in the combined blarcamesine group and 99% in the placebo group. Most enrolled participants would be characterized as early AD (Stage 3) [38] with baseline MMSE score 20–28, and the majority were on background therapy of cholinesterase inhibitors (ChEIs) and/or memantine to treat AD (Table 1). Baseline AD status was further supported by the elevated baseline levels of plasma p-Tau (181) and p-Tau (231), which confirmed AD pathology for participants, consistent with abnormal CSF amyloid-beta status in previous studies [39].

**Table 1:** Demographic characteristics of the Intent-to-Treat (ITT) population.

Demographic Characteristics	Blarcamesine 30 mg (*N* = 154)	Blarcamesine 50 mg (*N* = 144)	Blarcamesine Group (*N* = 298)	Placebo (*N* = 164)
Sex, n (%)				
Female	74 (48.1)	69 (47.9)	143 (48.0)	82 (50.0)
Male	80 (51.9)	75 (52.1)	155 (52.0)	82 (50.0)
Age, Mean (SD)	73.7 (6.6)	74.1 (6.3)	73.9 (6.5)	73.5 (6.3)
Race, n (%)				
Asian	3 (1.9)	4 (2.8)	7 (2.3)	2 (1.2)
Black or African American	0 (0.0)	0 (0.0)	0 (0.0)	2 (1.2)
Other	1 (0.6)	0 (0.0)	1 (0.3)	3 (1.8)
White	150 (97.4)	140 (97.2)	290 (97.3)	157 (95.7)
Ethnicity, n (%)				
Hispanic or Latino/a or of Spanish origin	5 (3.2)	2 (1.4)	7 (2.3)	1 (0.6)
Not Disclosed	7 (4.5)	6 (4.2)	13 (4.4)	8 (4.9)
Not Hispanic or Latino/a or of Spanish origin	142 (92.2)	136 (94.4)	278 (93.3)	155 (94.5)
APOE ε4 genotype, n (%)				
Noncarrier	47 (30.5)	47 (32.6)	94 (31.5)	46 (28.0)
Carrier	99 (64.3)	89 (61.8)	188 (63.1)	106 (64.6)
Heterozygotes	69 (44.8)	65 (45.1)	134 (45.0)	76 (46.3)
Homozygotes	30 (19.5)	24 (16.7)	54 (18.1)	30 (18.3)
Missing	8 (5.2)	8 (5.6)	16 (4.0)	12 (7.3)
Baseline clinical scores, Mean (SD)				
ADAS-COG13 score	28.4 (8.4)	28.9 (9.1)	28.6 (8.7)	30.4 (8.4)
ADCS-ADL score	66.7 (7.4)	67.0 (7.9)	66.9 (7.6)	66.4 (7.1)
CDR-SB score	3.8 (1.6)	3.8 (1.8)	3.8 (1.7)	4.1 (1.8)
MMSE score	23.6 (3.1)	23.6 (2.8)	23.6 (2.9)	23.0 (2.7)
Baseline CDR-Global scores, n (%)				
0	0 (0.0)	1 (0.7)	1 (0.3)	0 (0.0)
0.5	98 (63.6)	96 (66.7)	194 (65.1)	94 (57.3)
1.0	54 (35.1)	45 (31.3)	99 (33.2)	68 (41.5)
2.0	1 (0.6)	2 (1.4)	3 (1.0)	2 (1.2)
3.0	1 (0.6)	0 (0.0)	1 (0.3)	0 (0.0)
MMSE score at baseline, n (%)				
≤ 20	22 (14.3)	21 (14.6)	43 (14.4)	25 (15.2)
>20	132 (85.7)	123 (85.4)	255 (85.6)	139 (84.8)
Concomitant AD medication, n (%)				
Acetylcholinesterase inhibitors	102 (66.2)	104 (72.2)	206 (69.1)	108 (65.9)
Memantine	19 (12.3)	17 (11.8)	36 (12.1)	18 (11.0)
Baseline Plasma p-Tau (181)				
No. of participants evaluated at baseline	145	132	277	153
Baseline mean (SD), pg/mL	61.88 (25.44)	62.62 (25.75)	62.23 (25.54)	65.42 (28.05)
Baseline Plasma p-Tau (231)				
No. of participants evaluated at baseline	102	97	199	123
Baseline mean (SD), pg/mL	29.02 (29.55)	34.19 (50.76)	31.54 (41.24)	27.08 (34.58)

**Fig. 2:**
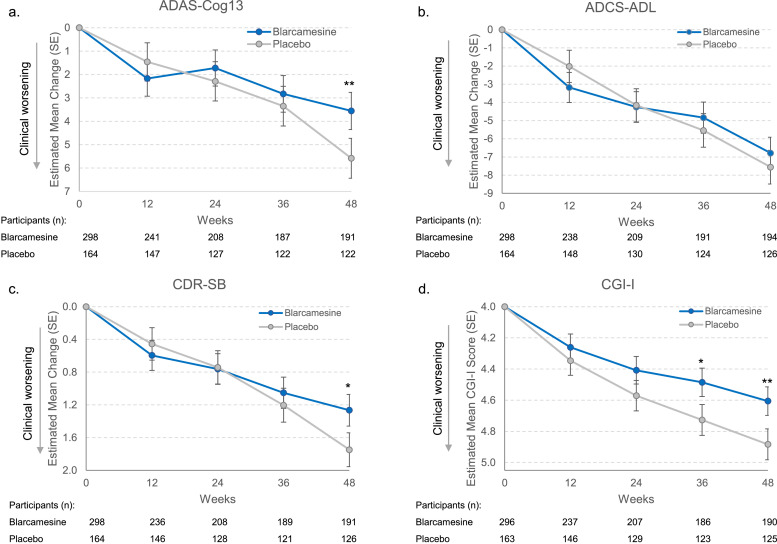
Clinical efficacy endpoints estimated mean change from baseline, blarcamesine versus placebo, ITT population.

Clinical endpoint results are reported in Table 2 in terms of improvement from baseline at Week 48, with the results per visit plotted in Fig. 2; results for assigned 30 mg and 50 mg groups are plotted in Supplemental Figure 1. For the primary endpoint ADAS-Cog13, blarcamesine group is significantly better than placebo (mean difference vs. placebo −2.027 [95%CI −3.522 to −0.533]; *P* = 0.008), representing a 36.3% reduction in clinical decline at 48 weeks. Similar results vs. placebo were observed for both 50 mg blarcamesine (difference of −2.149 [95%CI −3.979 to −0.319]; *P* = 0.021), representing a 38.5% reduction in clinical decline at 48 weeks; and for 30 mg blarcamesine dosage groups (difference of −1.934 [95%CI −3.639 to −0.228]; *P* = 0.026), representing a 34.6% reduction in clinical decline at 48 weeks. Co-primary endpoint ADCS-ADL improved for blarcamesine-treated patients relative to placebo but did not reach statistical significance at 48 weeks. The secondary endpoint CDR-SB was significantly improved for blarcamesine group vs. placebo (difference of −0.483 [95%CI −0.853 to −0.114]; *P* = 0.010), representing a 27.6% reduction in clinical decline at 48 weeks. Significant improvement from placebo was also observed for both 50 mg (difference of −0.465 [95%CI −0.918 to −0.012]; *P* = 0.045) and 30 mg (difference of −0.502 [95%CI −0.924 to −0.080]; *P* = 0.020) assigned dose groups. CGI-I was significantly improved in the active treatment group vs. placebo (difference of −0.278 [95% CI −0.466 to −0.089]; *P* = 0.004), as well as both 50 mg (difference of −0.314 [95%CI −0.545 to −0.082]; *P* = 0.008) and 30 mg (difference of −0.248 [95%CI −0.464 to −0.033]; *P* = 0.024) groups.

**Table 2:** Primary and secondary endpoints, Intent-to-Treat (ITT) population.

	Individual Group Comparison			Group Comparison	
	Blarcamesine 30 mg (*N* = 154)	Blarcamesine 50 mg (*N* = 144)	Placebo (*N* = 164)	Blarcamesine (*N* = 298)	Placebo (*N* = 164)
**Primary efficacy endpoints**					
Change from baseline to week 48 in the **ADAS-Cog13** score					
No. of participants at week 48	108	83	122	191	122
Adjusted mean change	3.650	3.436	5.584	3.555	5.582
Adjusted mean difference vs. placebo (95% CI)	−1.934 (−3.639 to −0.228)	−2.149 (−3.979 to −0.319)	..	−2.027 (−3.522 to −0.533)	..
P value vs. placebo	0.026*	0.021*	..	0.008**	..
Less decline, %	34.6%	38.5%	..	36.3%	..
Change from baseline to week 48 in the **ADCS-ADL** score					
No. of participants at week 48	109	85	126	194	126
Adjusted mean change	−6.702	−6.940	−7.592	−6.785	−7.560
Adjusted mean difference vs. placebo (95% CI)	0.890 (−0.992 to 2.772)	0.652 (−1.370 to 2.673)	..	0.775 (−0.874 to 2.423)	..
P value vs. placebo	0.354	0.527	..	0.357	..
Less decline, %	11.7%	8.6%	..	10.3%	..
**Secondary efficacy endpoint** Change from baseline to week 48 in the **CDR-SB** score					
No. of participants at week 48	107	84	126	191	126
Adjusted mean change	1.253	1.290	1.755	1.266	1.749
Adjusted mean difference vs. placebo (95% CI)	−0.502 (−0.924 to −0.080)	−0.465 (−0.918 to −0.012)	..	−0.483 (−0.853 to −0.114)	..
P value vs. placebo	0.020*	0.045*	..	0.010*	..
Less decline, %	28.6%	26.5%	..	27.6%	..
**Exploratory endpoint** Improvement from baseline to week 48 in the **CGI-I** score					
No. of participants at week 48	107	83	125	190	125
Adjusted improvement	4.634	4.568	4.882	4.606	4.883
Adjusted mean difference vs. placebo (95% CI)	−0.248 (−0.464 to −0.033)	−0.314 (−0.545 to −0.082)	..	−0.278 (−0.466 to −0.089)	..
P value vs. placebo	0.024*	0.008**	..	0.004**	..
Less decline, %	5.1%	6.4%	..	5.7%	..

Clinical efficacy endpoints were analyzed using mixed model for repeated measures (MMRM) estimates for the least-squares mean change from baseline at 12, 24, 36, and 48 weeks, with error bars representing standard error (SE). The number of trial participants with analyzed results at each visit is noted beneath the x axis. CGI-I baseline is represented as a score of 4, which represents “no change” in clinical improvement. Asterisks indicate statistically significant differences, where *: p value < 0·05,**: *p* < 0.01.

The relatively weaker effect of blarcamesine compared to placebo at the first time point (Week 12) is mostly related to initial tolerability caused by a relatively steep up titration and is most pronounced in ADCS-ADL and CDR-SB scores in the 50 mg dose groups (Supplemental Figure 1), suggesting there is a temporary functional weakening as patients adjust to higher doses during and after titration in the ITT population. Blarcamesine and placebo groups had 72 (75%) and 16 (57.2%) patient discontinuations in this early titration phase on or before Week 24, primarily due to TEAEs (Supplemental Table 2, Supplemental Figure 2). 40 (41.7%) blarcamesine patients and 5 (17.9%) placebo patients dropped out on or before the first analysis visit (Week 12). Even when early termination patients were excluded, the placebo group performed better than the blarcamesine group in these early phases (including Week 12) (Supplemental Figure 3). Therefore, there is no evidence that early termination will introduce a bias in favor of blarcamesine.

Consistent with a reduction of amyloid beta burden in the brain, plasma Aβ42/40 ratio increased significantly in blarcamesine-treated patients compared to placebo (mean difference vs. placebo (95% CI) of +0.013 (0.000 to 0.026), *P* = 0.048) with blarcamesine-treated patients increasing (+0.013) and placebo patients decreasing slightly (−0.0003) from baseline to week 48 (Supplemental Figure 4, Supplemental Table 3). Similarly, plasma levels of Nf-L, p-Tau (181), and p-Tau (231) all showed a smaller increase in blarcamesine-treated patients compared to placebo, although not reaching statistical significance (Supplemental Table 3).

Physical signs of neurodegeneration were also reduced in the blarcamesine treatment group, with structural MRI scans showing a significant reduction in whole brain grey matter volume loss, and corresponding decrease in the enlargement of lateral ventricles, in the active treatment group as well as both 30 mg and 50 mg treatment groups, compared with placebo (Supplemental Table 4, Supplemental Figure 5). Volume change of Whole Brain White Matter was not significantly different between treatment groups.

Clinical efficacy analysis of *SIGMAR1* gene variant (rs1800866) subgroups by MMRM demonstrated that variant non-carriers (common SIGMAR1 wild-type carriers; *n* = 199/101 blarcamesine/placebo) have a stronger response to blarcamesine treatment (Supplemental Tables 5 and 6) for ADAS-Cog13 (blarcamesine group vs. placebo difference of −2.317 [95% CI −4.182 to −0.453], 49.8% less decline, *P* = 0.015) and CDR-SB (blarcamesine group vs. placebo difference of −0.601 [95%CI −1.070 to −0.133], 33.7% less decline, *P* = 0.012) compared to the results for the *SIGMAR1* gene variant rs1800866 carrier subgroup (*n* = 87/58 blarcamesine/placebo): ADAS-Cog13 blarcamesine group vs. placebo (difference of −1.593 [95% CI −4.174 to 0.989], 25.2% less decline, *P* = 0.225); CDR-SB (difference of −0.230 [95% CI −0.826 to 0.367], 13.6% less decline, *P* = 0.449).

The tipping point analysis was performed under the missing not at random (MNAR) assumption (Supplemental Table 7). For ADAS-Cog13, placebo patients need to improve by 3.3 points, or blarcamesine patient worsening 1.9 points, from the imputed data under MAR to overturn the result of the primary analysis under MAR assumption. As the observed treatment difference is −1.973, this result supports the robustness of the MAR assumption in the primary analysis.

One death (0.6%) occurred in placebo group, and one death (0.3%) occurred in the blarcamesine group. No deaths were considered by the investigators to be related to assigned treatment. At least one serious AE occurred in 10.1% of the placebo group and in 16.7% of the blarcamesine group (Table 3). The proportion of participants with one or more treatment emergent AEs (TEAEs) was 76.8% in placebo group and 96.7% in blarcamesine group; the TEAEs were predominantly mild or moderate.

**Table 3:** Adverse events summary, full safety population.

Adverse Events Summary	Blarcamesine 30 mg	Blarcamesine 50 mg	Blarcamesine	Placebo
Patients, n	167	168	335	168
** **Death, n (%)	0	1 (0.6)	1 (0.3)	1 (0.6)
** **Death considered related to treatment	0	0	0	0
** **Participants with ≥1 Serious TEAEs, n (%)	25 (15.0)	31 (18.5)	56 (16.7)	17 (10.1)
TEAE, n (%)	159 (95.2)	165 (98.2)	324 (96.7)	129 (76.8)
** **TEAE leading to Treatment and Study Discontinuation, n (%)	41 (24.6)	67 (39.9)	108 (32.2)	12 (7.1)
Blarcamesine Titration AE ≥5.0%, n (%)	167	168	335	168
** **Dizziness	53 (31.7)	67 (39.9)	120 (35.8)	10 (6.0)
** **Confusional state	24 (14.4)	24 (14.3)	48 (14.3)	1 (0.6)
** **Balance disorder	12 (7.2)	13 (7.7)	25 (7.5)	1 (0.6)
** **Fatigue	9 (5.4)	10 (6.0)	19 (5.7)	0 (0)
** **Anxiety	8 (4.8)	10 (6.0)	18 (5.4)	0 (0)
** **Nausea	8 (4.8)	13 (7.7)	21 (6.3)	8 (4.8)
Blarcamesine Maintenance AE ≥5.0%, n (%)	148	153	301	161
** **Dizziness	28 (18.9)	48 (31.4)	76 (25.2)	9 (5.6)
** **Confusional state	16 (10.8)	24 (15.7)	40 (13.3)	4 (2.5)
** **Fall	12 (8.1)	9 (5.9)	21 (7.0)	16 (9.9)
** **Depressed mood	8 (5.4)	7 (4.6)	15 (5.0)	3 (1.9)
** **Headache	8 (5.4)	11 (7.2)	19 (6.3)	6 (3.7)
** **Anxiety	6 (4.1)	11 (7.2)	17 (5.6)	6 (3.7)
** **Balance Disorder	5 (3.4)	11 (7.2)	16 (5.3)	2 (1.2)

The most common blarcamesine AEs (5% or more) during treatment titration were dizziness (placebo 6.0%, blarcamesine 35.8%) and confusional state (placebo 0.6%, blarcamesine 14.3%) (Table 3). During treatment maintenance, the most common AEs were dizziness (placebo 5.6%, blarcamesine 25.2%) and confusional state (placebo 2.5%, blarcamesine 13.3%). The events of dizziness and confusional state were transient and predominantly mild to moderate (Grade 1 or 2). TEAEs led to treatment and study discontinuation in 7.1% of placebo and 32.2% of blarcamesine groups (Table 3). Early terminations in the active treatment group occurred predominantly before the first post-baseline scheduled analysis visit at Week 12 (Supplemental Table 2) mostly related to the relatively steep up titration to the respective target doses. No trend of serious or life-threatening adverse events was observed in the active treatment group.

## Discussion

In this Phase IIb/III randomized clinical trial, blarcamesine significantly slowed clinical progression at 48 weeks in the ITT population of participants with early AD for the cognitive primary endpoint ADAS-Cog13 and for the composite cognitive/functional secondary endpoint CDR-SB, while the co-primary endpoint ADCS-ADL did not reach statistical significance at Week 48. The co-primary outcome was met under the multiplicity control rule, since the differences in the least-squares mean (LSM) change from baseline to 48 weeks between the prespecified blarcamesine and placebo groups for ADAS-Cog13 was significant at a level of *P* < 0.025 and for CDR-SB was significant at a level of *P* < 0.025. In addition, current regulatory guidance from the FDA suggests that a sole cognitive endpoint is sufficient for demonstrating significance in early AD study populations [38]. In keeping with current regulatory practice, blarcamesine met the primary endpoint and should be considered a win as measured by ADAS-Cog13 at Week 48. The clinical effect of blarcamesine was supported by two independent biomarkers: a significant increase in pathological amyloid beta levels in plasma, representing a decrease in pathological amyloid beta in the brain, as well as a significant slowing in the rate of pathological brain atrophy in the brain as measured by MRI. Improvement in plasma Aβ42/Aβ40 ratio with blarcamesine treatment, as would be consistent with a reduction in amyloid beta in the brain is not entirely unexpected, as the Sigma-1 receptor and SIGMAR1 agonists are known to modulate the effects of amyloid precursor protein as well as amyloid-beta oligomers to reduce neurotoxicity [40]. In addition to the ability of blarcamesine to reduce cognitive impairments in amyloid beta AD models, blarcamesine significantly prevented amyloid beta-induced cognitive deficits with confirmed biomarker-responses in an animal model of AD [40].

All clinical endpoints demonstrated improvement in the blarcamesine treated group as well as the 30 mg and 50 mg blarcamesine groups at 48 weeks: general cognitive score (ADAS-Cog13), clinical dementia rating (CDR-SB), and global clinical improvement (CGI-I) all reached statistical significance, while the functional outcome ADCS-ADL improved but did not reach full significance. A possible explanation is that the ADCS-ADL scale is designed for AD with overt dementia and is less sensitive for early AD; recent studies comparing ADCS-ADL to other functional scoring outcomes suggest it may not be the most sensitive for early AD [41], and trials for donepezil, galantamine, and rivastigmine have all reported statistically significant differences in ADCS-ADL vs. placebo for subjects with moderate-to-severe AD but did not observe any significant differences in mild AD [42]. At 48 weeks, blarcamesine group demonstrated numerically superior clinical efficacy compared with recent anti-amyloid therapies even within a shorter treatment duration; ADAS-Cog13 difference of −2.027 at 48 weeks vs. −1.35 reported for Kisunla/donanemab at 76 weeks [6], and CDR-SB difference of −0.483 at 48 weeks vs. −0.451 reported for Leqembi/lecanemab at 72 weeks [7]. Recent regulatory actions on anti-amyloid mAb drug trials [5], [6], [7] were made with CDR-SB serving as the sole primary endpoint; when assessing CDR-SB, blarcamesine demonstrates significant improvement over placebo in the active treatment group as well as both the 30 mg and 50 mg dosage groups. The physician-evaluated global endpoint Clinical Global Impression – Improvement (CGI-I) also demonstrated significant improvement over placebo at 48 weeks in the active treatment group as well as both 30 mg and 50 mg blarcamesine dosage groups, as further support of efficacy in this study population. Taken as a whole, the clinical endpoints demonstrate efficacy based on current regulatory standards for early AD, and the magnitudes of the clinical effects are numerically superior to recently approved therapies for early AD.

Blarcamesine treatment was associated not only with slowing of progression in cognitive decline but also with amelioration of key indicators of AD pathology, namely increase in plasma Aβ42/Aβ40 ratio and reduction in brain volume loss. Plasma Aβ42/40 ratio has been consistently shown to be a reliable measure for amyloid plaque deposition [43] and so a substantial increase in plasma Aβ42/40 ratio is a strong indicator that amyloid plaque burden may be decreasing in blarcamesine-treated patients. This current clinical study has now strengthened the previously reported [40] link between blarcamesine and AD pathophysiology. Taken together, these results suggest a potential relationship between blarcamesine treatment and plasma levels of these proteins, as well as the A/T/N framework for AD pathology.

The results of the prespecified *SIGMAR1* gene variant subgroup analysis reinforce the previously confirmed (from the earlier published Phase 2a AD study [18]) mechanism of action for blarcamesine in AD, beneficial clinical effect through upstream SIGMAR1 activation. Compared to the full ITT population, subjects *without* the mutated SIGMAR1 rs1800866 variant (common SIGMAR1 wild-type carriers; *n* = 199/101 blarcamesine/placebo) treated with blarcamesine experienced a greater clinical benefit for both ADAS-Cog13 (slowed clinical progression by 49.8% vs. 36.3%) and CDR-SB (slowed clinical progression by 33.7% vs. 27.6%). Conversely, the subgroup of subjects carrying the *SIGMAR1* rs1800866 mutation (*n* = 87/58 blarcamesine/placebo) who were treated with blarcamesine did not reach significance in any reported clinical endpoints relative to placebo. The confirmed *SIGMAR1* gene variant data might allow the possibility of utilizing the *SIGMAR1* rs1800866 SNP as a stratification biomarker (enriching common *SIGMAR1* wild-type carriers by excluding *SIGMAR1* rs1800866 mutation carriers) effectively to stratify patients within the precision medicine paradigm.

The study had some missing data. 45 out of 462 ITT patients discontinued on or before reaching Week 12, the first analysis visit. Among these patients, 40 were in the blarcamesine group, and 36 dropped out due to TEAEs. The missing data in the study dropouts were primarily due to patients who did not tolerate the relatively short and steep titration schedule of this study. There is no evidence that these patients introduced a bias in favor of the blarcamesine group by dropping out early. Going forward, the titration schedule can be adjusted to slower titration and lower target dose.

To our knowledge this is the first report of a therapeutic agent for AD that has demonstrated an attenuation in global brain volume loss measured by MRI and reduction of the expansion of the lateral ventricular volume compared to placebo. Volumetric MRI improvements associated with blarcamesine appeared global and may be in response to restoration of cellular homeostasis [14]. The global improvements in volumetric MRI associated with blarcamesine are accompanied by reducing the decline of clinical disease progression, which suggests the drug effects might be exerted by mitigating neurodegeneration. In contrast, anti-amyloid beta monoclonal antibodies have been associated with amyloid-related imaging abnormalities-edema (ARIA-E), amyloid-related imaging abnormalities-hemorrhages (ARIA-H) and a decrease in whole brain volume, i.e. brain atrophy (ARIA-A) compared with placebo as well as decreases in other brain regions and a mean increase in ventricular volume compared with placebo [12], [44].

Blarcamesine was relatively safe in the study population, with no trends of severe or life-threatening and with no associated neuroimaging adverse events. There were no deaths attributable to blarcamesine or placebo. The initially observed early discontinuations and adverse events might be related to the timing of the up titration of blarcamesine to the target doses coupled with administration at consistent timepoints relatively early in the morning as specified in the protocol. These events can likely be addressed by changing administration to nighttime dosing, as has been positively observed in the compassionate use program of blarcamesine administration coupled with once daily oral dosing without requiring reaching the higher target doses. Further evaluation on management and reduction of TEAE occurrence will be important.

This study has some limitations. First, there was variability in total blarcamesine doses received and/or duration of blarcamesine dosing. Second, data collection was for 48 weeks, limiting long-term understanding of blarcamesine; however, a 96-week OLE extension study (ATTENTION-AD) followed. Third, the studied populations were primarily White (96.8%), which may limit generalizability to other populations due to a lack of racial and ethnic diversity. In order to demonstrate effectiveness in a broader population, future studies will require a more diversified patient cohort. Fourth, although no related protocol amendments were necessary, this trial was conducted during the COVID-19 pandemic. Finally, non-significance of the functional measure ADCS-ADL at 48 weeks is considered to be due to the relatively low sensitivity of the scale in an early AD population and the relatively short duration of the study.

Blarcamesine, a small molecule administered orally once daily, has numerically superior clinical efficacy to approved therapies while also slowing neurodegeneration in early AD patients. Blarcamesine has a demonstrated safety profile and does not require routine MRI monitoring, and given its differentiated mechanism of action, could represent a novel treatment that is complementary or an alternative to the anti-beta amyloid drugs.

## Author contributions

All authors made contributions to data acquisition, analysis, or interpretation and critically revised and approved the manuscript.

## Funding

This work was funded by Anavex Life Sciences.

## Trial registration

Clinicaltrials.gov Identifier: NCT03790709.

## Role of funder/support

Anavex Life Sciences was responsible for design and conduct of the trial; collection, management, analysis, and interpretation of the data; preparation, review, or approval of the manuscript; and decision to submit the manuscript for publication.

## Additional contributions

We thank all the trial participants and their families and caregivers who participated in the ANAVEX2-73-AD-004 trial as well as the site staff, raters, and site investigators, members of the data and safety monitoring board.

## Submission category

Research Article. Randomized clinical trial report.

## Declaration of competing interest

DISCLOSURES: Dr. Sabbagh discloses ownership interest (stock or stock options) in NeuroTau, Inc., uMETHOD, Athira Pharma, Inc., and CervoMed and Lighthouse Pharmaceuticals; consulting for Alzheon, Inc, Genentech (Roche Group), Prothena, Novo Nordisk, Anavex Life Sciences, T3D Therapeutics, Inc., Eisai Co., Ltd., Eli Lilly and Co., and KeifeRx.

Dr. Macfarlane has received paid honoraria from the following pharmaceutical companies for various speaking engagements and advisory board services: Eisai, Eli Lilly, Janssen-Cilag, Lundbeck, Novo Nordisk. Dr. Macfarlane is contracted by Anavex Life Sciences to provide medical monitoring services for Anavex's Rett syndrome studies.

Dr. Grimmer received consulting fees from AbbVie, Alector, Anavex Life Sciences, Biogen, Cogthera, Eli Lilly, Functional Neuromodulation, Grifols, Iqvia, Janssen, Noselab, Novo Nordisk, NuiCare, Orphanzyme, Roche Diagnostics, Roche Pharma, UCB, and Vivoryon; lecture fees from Biogen, Eisai, Grifols, Medical Tribune, Novo Nordisk, Roche Pharma, Schwabe, and Synlab; and has received grants to his institution from Biogen, Eisai, and Roche Diagnostics.

Dr. O'Brien's institution has received consultancy and/research funding from Anavex Life Sciences, Eisai, UCB Pharma, ES Therapeutics, Kinoxis Pharmaceuticals, Supernus, Autobahn, Shanghai Zhimeng, Epidarex, and government grant funding from NHMRC (APP1176426), MRFF, DoD and NINDS.

Dr. Woodward has received honoraria for speaking and expert advice from Actinogen, Biogen, Roche, MSD/Merck, Glaxo Smith Kline, Cognition Therapies, Eisai, Novo Nordisk and Pfizer. He was previously paid for his role as Chief National Investigator for Anavex Life Sciences. He owns no shares and has no direct employment with any pharmaceutical company or Biotech.

Dr. Tartaglia is SAB member of Brain Injury Canada, PSP Awareness, and Women's Brain Project.

Advisory to Eisai, Eli Lilly and QurAlis and received Grant funding from NIH, Weston Brain Institute, Tanenbaum Institute for Science in Sport and participated in clinical trials: Biogen, Novo Nordisk, Janssen, Roche, Anavex Life Sciences, Passage Bio, Green Valley.

Dr. Frank received paid honoraria from the following pharmaceutical companies for advisory board services: Eisai, Eli Lilly, Roche Pharma, Novo Nordisk.

Dr. Lai has received a paid honorarium for speaking engagements with INmune Bio.

Dr. Lewis is supported by a National Health and Medical Research Council Leadership Fellowship (1195830) and has received research funding from The Michael J. Fox Foundation and the Australian Research Council, as well as consulting for Pharmaxis Ltd.

Dr. Kurrle has received honoraria for educational activities from Roche Diagnostics and Novartis.

Dr. Cohen discloses consulting work (no personal fees received) for: Alnylam, Biogen, Biohaven, Cassava, Cogstate, Cognivue, Eisai, Eli Lilly, INmune Bio, Novo Nordisk, ProMIS Neuroscience, Roche, RetiSpec, SciNeuro; and research grants (paid to institution only) from: AbbVie, AgeneBio, Alector, Alnylam, Alzheon, Anavex Life Sciences, Biogen, Cassava, Eisai, Eli Lilly, Janssen, Novo Nordisk, Roche, RetiSpec, UCB Biopharma.

Dr. Grunfeld has received paid honoraria from the Janssen-Cilag for advisory board services.

Dr. Morris has no financial conflicts of interest to declare.

Dr. Connell does not have any professional conflicts of interest.

Dr. Thompson does not have any conflicts of interests to declare.

Dr. Tacik does not have any conflicts of interests to declare.

Dr. Perry has received paid honoraria from the following pharmaceutical companies for various speaking engagements and advisory board services: Eisai, Eli Lilly, MSDF, Biogen, and Roche.

Dr. Sharif does not have any conflicts of interest to disclose.

Dr. Kalafatis does not have any conflicts of interests to declare.

Dr. Munisamy does not have any conflicts of interests to declare.

Dr. Pearson has received paid honoraria for speaking and advice from Biogen, Eli Lilly and Boehringer-Ingelheim.

Dr. Sturm does not have any conflicts of interests to declare.

Dr. Oschmann received research support as well as speaking fees and travel fees from Alexion, Bayer Health Care, Biogen, Janssen, Merck Serono, Novartis, Pfizer, Roche, Sanofi Genzyme, TEVA.

Dr. Hsiung discloses that he has received grants or contracts from CIHR, NIA/NIH and has participated in expert advisory committee supported by Biogen, Roche, and NovoNordisk. Dr. Hsiung is the current president of C5R (Consortium of Canadian Centres for Clinical Cognitive Research).

Dr. Lynch does not have any conflicts of interests to declare.

Dr. Brew does not have any conflicts of interests to declare.

Dr. Tucker is employed by Anavex Life Sciences as an independent consultant to provide medical monitoring services for the Alzheimer's disease program.

Dr. Ingram discloses no financial ownership interest in any pharmaceutical company but has been paid honoraria by Eisai, Merck, Biogen, Roche, Janssen, Eli Lilly to participate in health care planning and messaging regarding their products’ impact on dementia. Anavex research responsibilities were contractually held by Kawartha Centre ∼ Redefining Healthy Aging, previously owned by Dr. Ingram. This company has changed ownership as of January 5, 2023.

Dr. Pasternak has received grant support to his institution and hold shares in Zywie Bio LLC. He has received speakers fees from Eli Lilly.

Dr. MacSweeney does not have any conflicts of interests to declare.

Dr. Short has received paid honoraria from Roche and Eisai for Advisory Board services and speaking engagements.

Dr. Bhatt does not have any conflicts of interests to declare.

Dr. Drysdale discloses that he has been paid for conducted research by the following companies, Eli Lilly, Cassava Sciences, Roche, Anavex Life Sciences, Lundbeck and Biogen.

Dr. Mannering does not have any conflicts of interests to declare.

Dr. Henri-Bhargava has received paid honoraria for Advisory boards / speaking engagements for Roche, Lilly, Eisai, Boehringer Ingelheim; Clinical trial payments from: Lilly, Roche, Boheringer Ingelhiem, Anavex Life Sciences, Cerevel, Green Valley Shanghai, Intelgenx; Grants from Canadian Consortium on Neurodegeneration in Aging, Centre for Aging and Brain Health Innovation, Manning Cognitive Health Initiative.

Dr. Froelich has received honoraria for consulting or presentations from Biogen, BioVie, Eisai, Grifols, Janssen Cilag, Neurimmune, Noselab, NovoNordisk, Roche, TauRX, Schwabe; Honoraria for Clinical study committees from Avanir/Otsuka, PharmatrophiX, Charité Berlin, Neuroscios, Vivoryon; Clinical trials (honoraria to his institution) from Axon Neuroscience, Anavex Life Sciences, Alector, Boehringer Ingelheim, Eisai, Hummingbird, NovoNordisk, Noselab.

Dr. Chertkow has been supported by a Foundation Grant from the CIHR (Canadian Institutes for Health Research), along with funding from the National Institute of Health (US), the Weston Foundation and the Baycrest Health Sciences Foundation. He has participated as a site PI in pharmaceutical trial activities sponsored by Hoffmann-La Roche, TauRx, Lilly, Anavex Life Sciences, Alector, Biogen, Esai, and Immunocal (site investigator for trials). He has participated as an unpaid advisor in 2020 for establishment of an international database by Biogen. He has participated in advisory boards for Esai and Lilly Co., with honoraria going to the Rotman Research Institute. He is Scientific Director for the CCNA, which receives partner support from partners including Pfizer, Lilly, Sanofi.

Dr. Mander does not have any conflicts of interests to declare.

Dr. Wiltfang does not have any conflicts of interests to declare.

Dr. Prins performed consultancy work for Aribio, Amylyx, Eli-Lilly and Janssen and received a speaker fee from Biogen. He is co‐PI of of a current trial with Fuji Film Toyama Chemical. He is CEO and co‐owner of Brain Research Center, the Netherlands.

Dr. Peters received consulting or lecture fees from Biogen, Eisai, Eli Lilly, Grifols, Medical Tribune, Noselab, Novo Nordisk, Prinnovation, Priavoid, Roche Diagnostics and Roche Pharma; and has received grants to his institution from Biogen, Eisai, Eli Lilly, Noslab, Predemtec, Roche Pharma, Roche Diagnostics and Vivoryon.

Dr. Smith has received personal consulting fees from Alnylam Pharmaceuticals and Eli Lilly.

Dr. Dautzenberg has participated as PI in pharmaceutical trials activities sponsored by TauRx, Lilly, Anavex Life Sciences, Alector, Biogen Boehringer Ingelheim, Eisai, NovoNordisk, Green Valley Shanghai, Roche and received a speaker fee from NovoNordisk as National PI.

Dr. Evans does not have any conflicts of interests to declare.

Dr. Villa does not have any conflicts of interests to declare.

Dr. Gordon does not have any conflicts of interests to declare.

Dr. Jubault does not have any conflicts of interests to declare.

Dr. Guizard does not have any conflicts of interests to declare.

Dr. Kaufmann discloses being an employee of and ownership interest (stock or stock options) in Anavex Life Sciences.

Dr. Kun Jin discloses being an employee of and ownership interest (stock or stock options) in Anavex Life Sciences.

Dr. Chezem discloses being an employee of and ownership interest (stock or stock options) in Anavex Life Sciences.

Dr. Missling discloses being an employee of and ownership interest (stock or stock options) in Anavex Life Sciences.

Dr. Babajide does not have any conflicts of interest to declare.

Dr. Brodtmann has received paid honoraria from the following pharmaceutical companies for advisory board services: Biogen, Roche and Eisai.

Dr. Asher does not have any conflicts of interests to declare.

## Appendix Supplementary materials

Image, application 1

Image, application 2
