# Supplementary material for: Blarcamesine for the treatment of Early Alzheimer's Disease: Results from the ANAVEX2-73-AD-004 Phase IIB/III trial
Source: J Prev Alzheimers Dis. 2025 Jan 1;12(1):100016. doi: 10.1016/j.tjpad.2024.100016 (PMC12184016; doi:10.1016/j.tjpad.2024.100016)
Supplement: Supplementary file 1 [file mmc1.pdf]

Supplementary Figures

DRAFT - CONFIDENTIAL

Supplementary Figure 1. Clinical efficacy endpoints estimated mean change from baseline, blarcamesine versus placebo, assigned treatment groups, ITT population.

a.

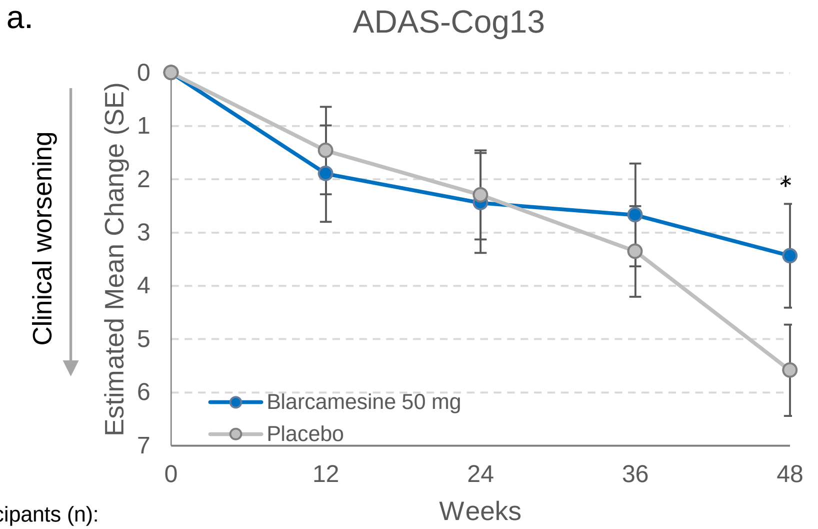

Participants (n):					
Blarcamesine 50 mg	144	111	91	79	83
Placebo	164	147	127	122	122

b.

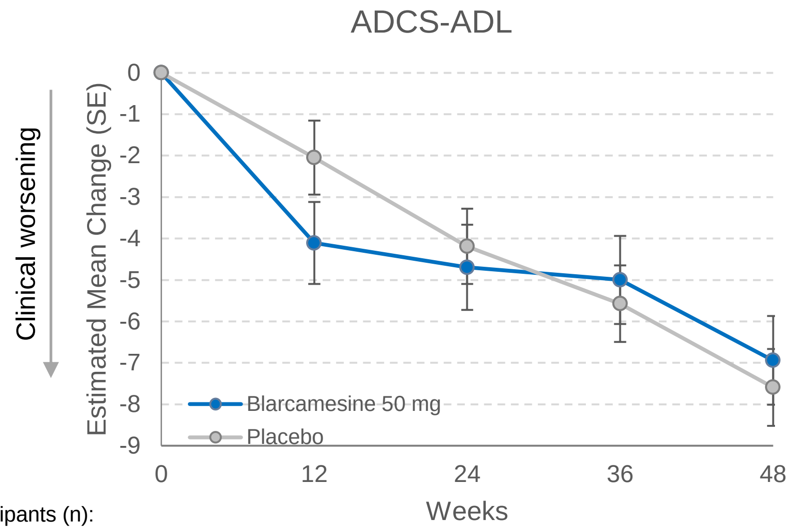

Participants (n):					
Blarcamesine 50mg	144	111	91	82	85
Placebo	164	148	130	124	126

c.

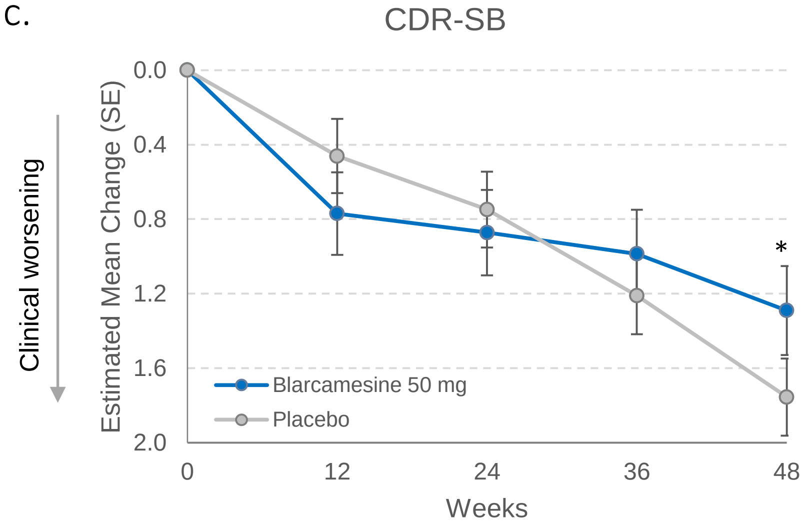

Participants (n):					
Blarcamesine 50mg	144	108	91	81	84
Placebo	164	146	128	121	126

d.

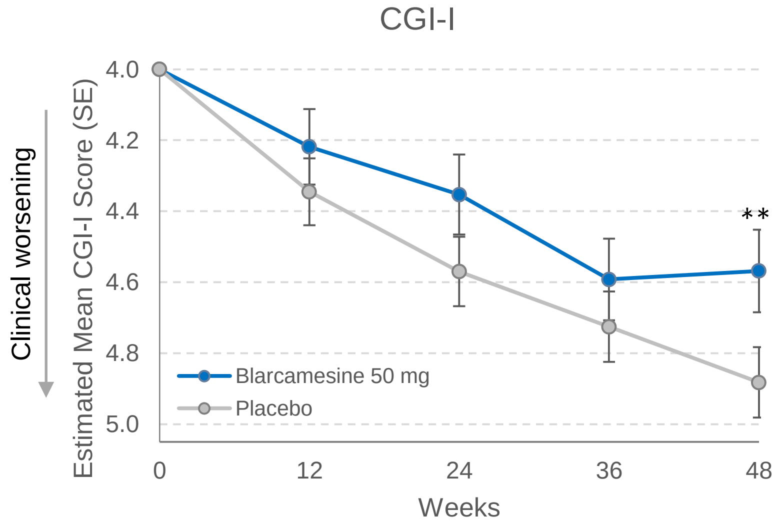

Participants (n):					
Blarcamesine 50mg	143	107	89	82	83
Placebo	163	146	129	123	125

Clinical efficacy endpoints were analyzed using mixed model for repeated measures (MMRM) estimates for the least-squares mean change from baseline at 12, 24, 36, and 48 weeks, with error bars representing standard error (SE). The number of trial participants with analyzed results at each visit is noted beneath the x axis. CGI-I baseline is represented as a score of 4, which represents "no change" in clinical improvement. Asterisks (*) indicate statistically significant difference vs. placebo where p value < 0·05. **: p<0.01

DRAFT - CONFIDENTIAL

Supplementary Figure 1. Clinical efficacy endpoints estimated mean change from baseline, blarcamesine versus placebo, assigned treatment groups, ITT population.

e.

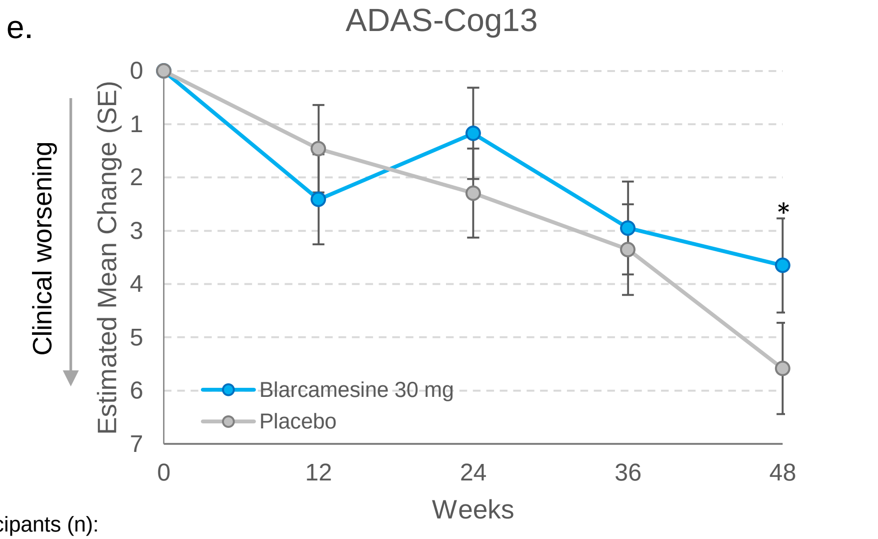

Participants (n):					
Blarcamesine 30mg	154	130	117	108	108
Placebo	164	147	127	122	122

f.

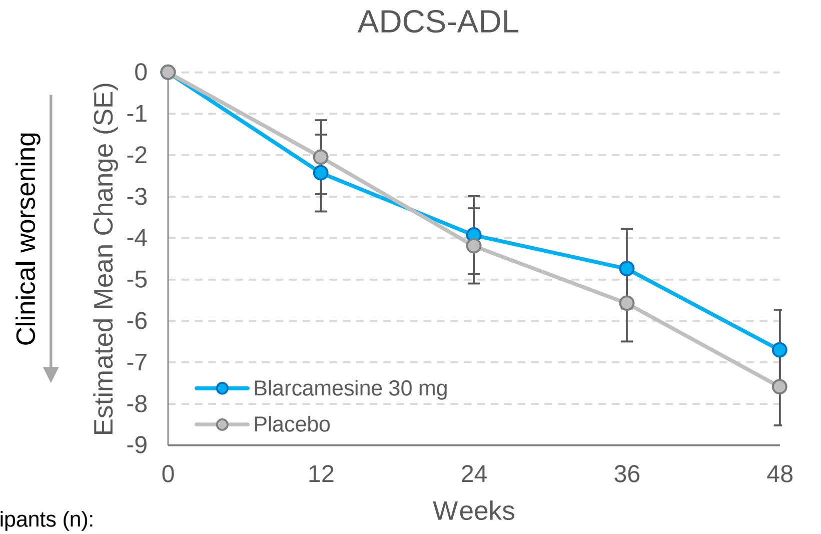

Participants (n):					
Blarcamesine 30mg	154	127	118	109	109
Placebo	164	148	130	124	126

g.

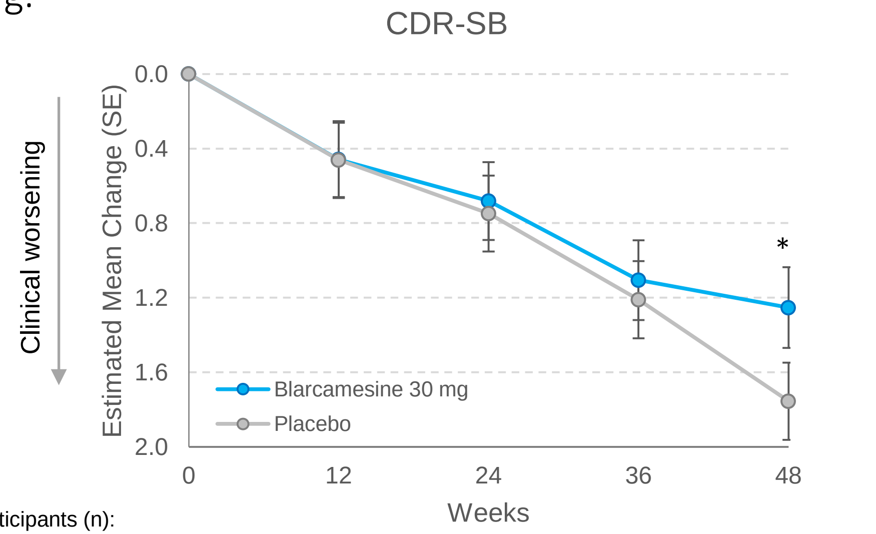

Participants (n):					
Blarcamesine 30mg	154	128	117	108	107
Placebo	164	146	128	121	126

h.

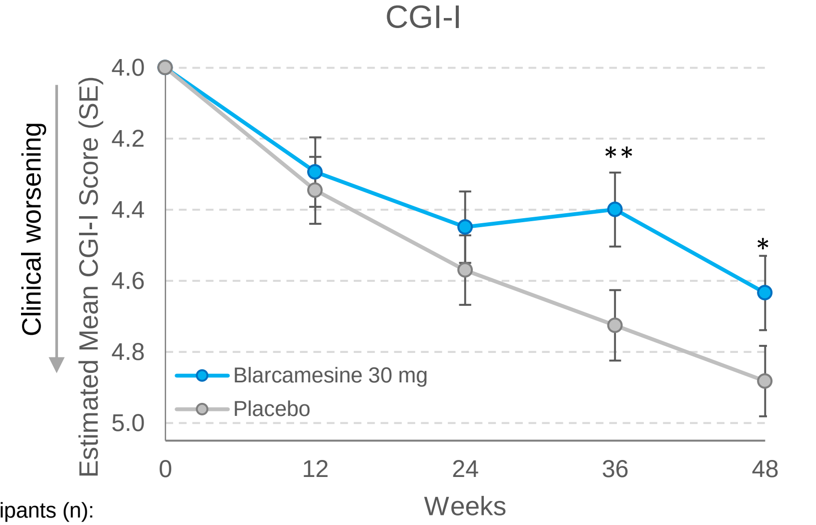

Participants (n):					
Blarcamesine 30mg	153	130	118	104	107
Placebo	163	146	129	123	125

Clinical efficacy endpoints were analyzed using mixed model for repeated measures (MMRM) estimates for the least-squares mean change from baseline at 12, 24, 36, and 48 weeks, with error bars representing standard error (SE). The number of trial participants with analyzed results at each visit is noted beneath the x axis. CGI-I baseline is represented as a score of 4, which represents “no change” in clinical improvement. Asterisks (*) indicate statistically significant differences from placebo where p value < 0·05. **: p<0.01

DRAFT - CONFIDENTIAL

Supplementary Figure 2. Early termination discontinuation reason by randomized treatment group, ITT population

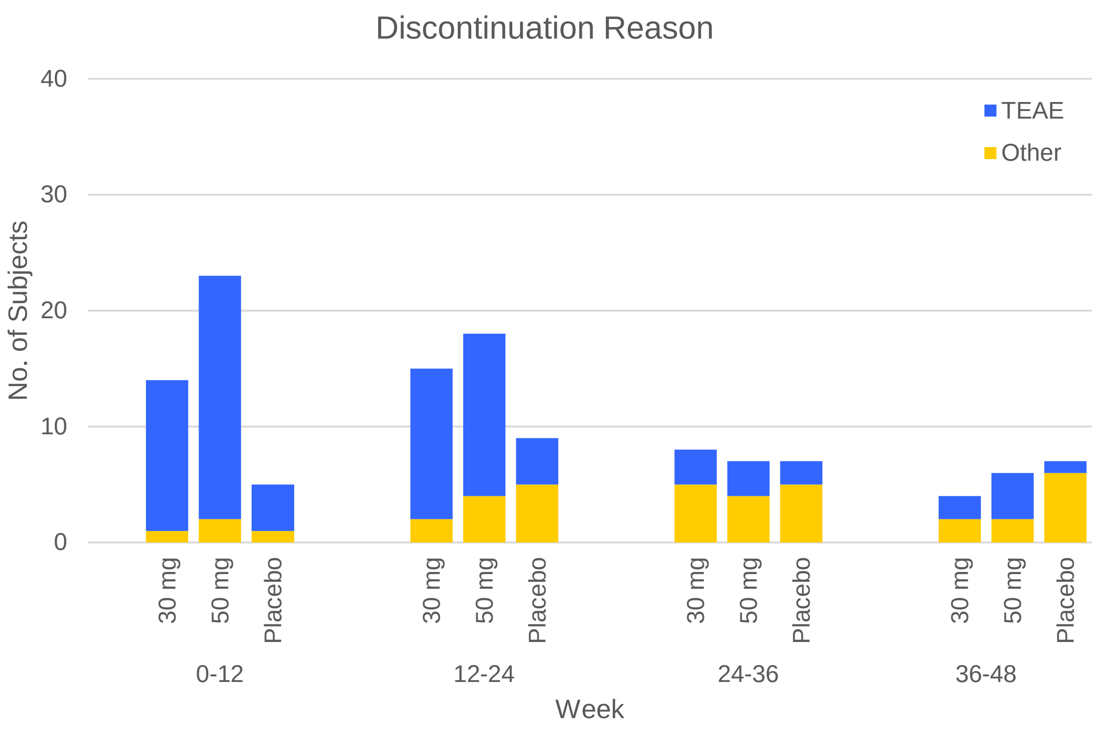

DRAFT - CONFIDENTIAL

Supplementary Figure 3. Clinical endpoint scores, ITT population excluding post week 12 dropouts.

a.

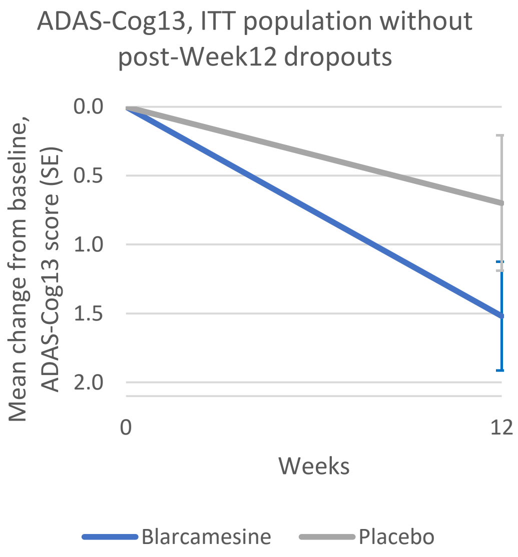

b.

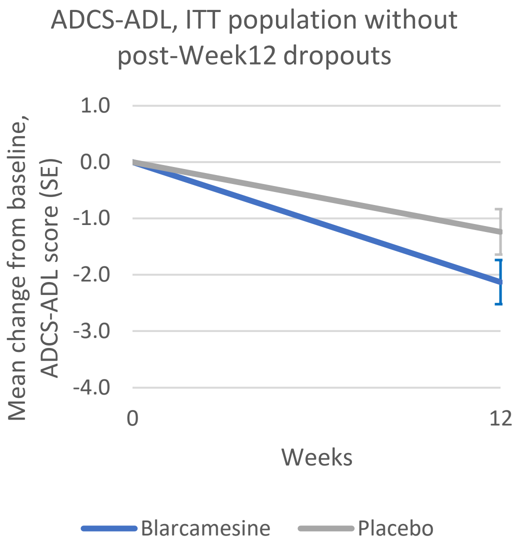

c.

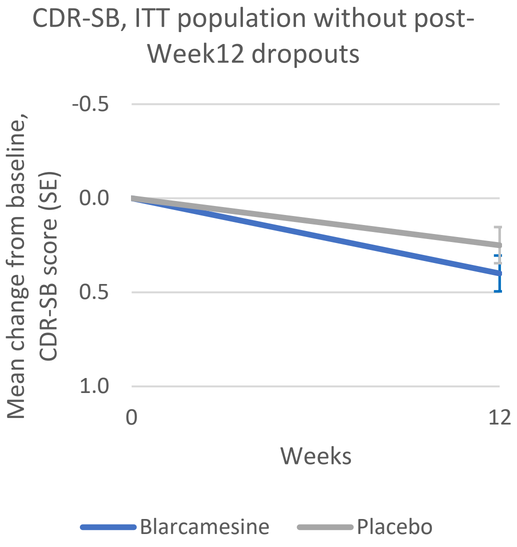

Supplementary Figure 4. Change in plasma Aβ42/40 Ratio at 48 weeks, blarcamesine vs. placebo.

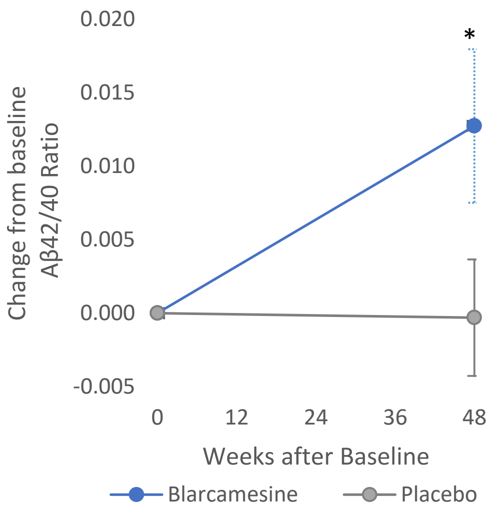

[caption] Change in plasma Aβ42/40 Ratio at 48 weeks after baseline, as determined by ELISA measurement. Results are mean change and error bars represent SE. (*) indicates statistically significant differences between treatment groups as determined by Welch two-tailed T-test, where $P < 0.05$.

Supplementary Figure 5. Annualized percent change in volumetric MRI at 48 weeks, blarcamesine pooled vs. placebo.

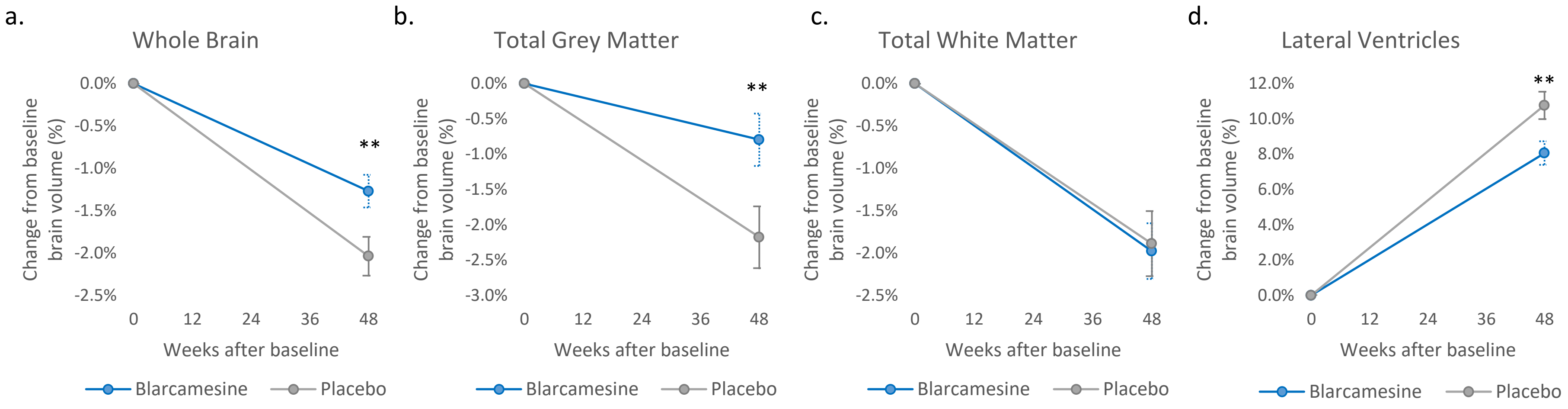

[Figure caption] Annualized change in brain volume at 48 weeks after baseline was calculated based on volumetric MRI scans for patients taken at baseline and 48 weeks/end of study. Reported values are for whole brain, total brain white/grey matter, and lateral ventricles, comparing pooled treatment and placebo groups. Results are based on linear modeling using treatment group, baseline volume, and baseline MMSE status as covariates. Statistical significance is indicated by asterisks [*, **, ***, ****] in cases where $p < 0·05, 0·01, 0·001$, or $0·0001$, respectively. (*-****) [end caption]
