# Supplementary material for: Blarcamesine for the treatment of Early Alzheimer's Disease: Results from the ANAVEX2-73-AD-004 Phase IIB/III trial
Source: J Prev Alzheimers Dis. 2025 Jan 1;12(1):100016. doi: 10.1016/j.tjpad.2024.100016 (PMC12184016; doi:10.1016/j.tjpad.2024.100016)
Supplement: Supplementary file 2 [file mmc2.docx]

**Supplementary Table 1. Accumulated dose exposure to blarcamesine of 30 mg and 50 mg target groups by study visit, ITT population.**

| **Treatment Group** | **Statistics** | **Week 12** | **Week 24** | **Week 36** | **Week 48** |
| --- | --- | --- | --- | --- | --- |
| Blarcamesine 30 mg | n | 154 | 123 | 116 | 113 |
|  | Mean (SD) | 879·0 mg (392·02) | 3032·6 mg (801·30) | 5166·1 mg (1259·72) | 7432·4 mg (1825·59) |
|  |  |  |  |  |  |
| Blarcamesine 50 mg | n | 143 | 100 | 96 | 92 |
|  | Mean (SD) | 825·1 mg (512·89) | 3273·2 mg (1435·52) | 5729·5 mg (2493·16) | 8110·2 mg (3501·94) |
|  |  |  |  |  |  |

Statistics of accumulated exposure to blarcamesine per study visit period in 30 mg/50 mg assigned target dosage groups in the ITT population at the time of each analysis visit.

**Supplementary Table 2. Early discontinuations, by scheduled visit period, ITT population**

| **Weeks** | **Blarcamesine**  **(N = 96),**  **n Disc. (%), n TEAE** | **Placebo**  **(N = 28),**  **n Disc. (%), n TEAE** |
| --- | --- | --- |
| 0-12 | 40 (41.7%), 36 | 5 (17.9%), 4 |
| 13-24 | 32 (33.3%), 27 | 11 (39.3%), 5 |
| 25-36 | 16 (16.7%), 5 | 7 (25%), 1 |
| 37-48 | 8 (8.3%), 6 | 5 (17.9%), 1 |

Summary of early discontinuation patients in the ITT population, with number and percentage of discontinuations at each visit period and number of TEAE related discontinuations. Designation of early discontinuation visit period is based on the final analysis visit with efficacy results for each patient.

**Supplementary Table 3. Plasma biomarker levels, change from baseline at 48 weeks, ITT population**

| Plasma Biomarker Endpoints | Blarcamesine 30 mg  (n = 154) | Blarcamesine 50 mg  (n =144) | Blarcamesine Group  (n = 298) | Placebo  (n = 164) |
| --- | --- | --- | --- | --- |
| Change from baseline to week 48, Aβ42/40 ratio | | | | |
| No. of participants evaluated | 67 | 52 | 119 | 78 |
| Mean change (SD) | +0.006 (0.028) | +0.021 (0.080) | +0.013 (0.057) | −0.0003 (0.035) |
| Mean difference vs. placebo (95% CI) | 0.007 (-0.004 to 0.017) | 0.021 (-0.002 to 0.045) | 0.013 (0 to 0.026) | -- |
| P value vs. placebo | 0.206 | 0.075 | 0.048* | -- |
| Change from baseline to week 48, Nf-L | | | | |
| No. of participants evaluated | 99 | 83 | 182 | 122 |
| Mean change (SD), pg/mL | +1.38 (6.54) | +1.96 (6.88) | +1.65 (6.68) | +4.92 (33.00) |
| Mean difference vs. placebo (95% CI) | -3.54 (-9.59 to 2.51) | -2.96 (-9.05 to 3.13) | −3.28 (−9.27 to 2.72) | -- |
| P value vs. placebo | 0.25 | 0.34 | 0.28 | -- |
| Change from baseline to week 48, p-Tau (181) | | | | |
| No. of participants evaluated | 92 | 73 | 165 | 117 |
| Mean change (SD), pg/mL | +2.92 (14.17) | +5.17 (16.33) | +3.92 (15.16) | +10.85 (85.63) |
| Mean difference vs. placebo (95% CI) | -7.92 (-23.86 to 8.02) | -5.67 (-21.78 to 10.44) | -6.93 (-22.77 to 8.92) | -- |
| P value vs. placebo | 0.33 | 0.49 | 0.39 | -- |
| Change from baseline to week 48, p-Tau (231) | | | | |
| No. of participants evaluated | 50 | 55 | 105 | 83 |
| Mean change (SD), pg/mL | +2.27 (13.54) | -1.51 (12.08) | +0.29 (12.87) | +3.86 (40.66) |
| Mean difference vs. placebo (95% CI) | -1.59 (-11.21 to 8.04) | -5.37 (-14.79 to 4.05) | -3.57 (-12.77 to 5.63) | -- |
| P value vs. placebo | 0.74 | 0.26 | 0.44 | -- |

Plasma samples were taken at baseline and 48 weeks for quantification of protein biomarkers. Statistical significance was evaluated by an unpaired two-tailed t-test comparison of the two groups, with a significance threshold of P < 0.05 (*). Patient numbers in each treatment group represent patients with both baseline and end of study results for each measurement; for Aβ42/40 ratio the number includes patients with baseline and end of study results for both Aβ42 and Aβ40.

**Supplementary Table 4. Volumetric MRI measurements, annualized percent change from baseline, general linear model, ITT population**

| MRI Measurement | | Individual Group Comparison | | | Combined Group Comparison | |
| --- | --- | --- | --- | --- | --- | --- |
|  | | Blarcamesine  30 mg  (n = 154) | Blarcamesine  50 mg  (n =144) | Placebo  (n = 164) | Blarcamesine Group  (n = 298) | Placebo  (n = 164) |
| Whole Brain, %change from baseline | |  |  |  |  |  |
|  | Number of subjects | 93 | 72 | 100 | 165 | 100 |
|  | Adjusted mean change (SE) | -1.14% (0.23) | -1.45% (0.26) | -2.04% (0.23) | -1.27% (0.19) | -2.04% (0.23) |
|  | Adjusted mean difference vs. placebo (95% CI) | 0.9% (0.35 to 1.44) | 0.59% (0.00 to 1.18) |  | 0.77% (0.29 to 1.25) | -- |
|  | P value vs. placebo | 0.001** | 0.049* |  | 0.002** | -- |
| Total Grey Matter, %change from baseline | |  |  |  |  |  |
|  | Number of subjects | 94 | 72 | 100 | 166 | 100 |
|  | Adjusted mean change (SE) | -0.62% (0.44) | -1.05% (0.50) | -2.18% (0.44) | -0.80% (0.37) | -2.18% (0.44) |
|  | Adjusted mean difference vs. placebo (95% CI) | 1.56% (0.52 to 2.61) | 1.13% (0.00 to 2.26) |  | 1.38% (0.46 to 2.3) | -- |
|  | P value vs. placebo | 0.003* | 0.049* |  | 0.004** | -- |
| Total White Matter, %change from baseline | |  |  |  |  |  |
|  | Number of subjects | 93 | 72 | 100 | 165 | 100 |
|  | Adjusted mean change (SE) | -2.01% (0.39) | -1.94% (0.44) | -1.89% (0.39) | -1.98% (0.33) | -1.89% (0.38) |
|  | Adjusted mean difference vs. placebo (95% CI) | -0.12% (-1.04 to 0.81) | -0.05% (-1.05 to 0.95) |  | -0.09% (-0.9 to 0.73) | -- |
|  | P value vs. placebo | 0.806 | 0.919 |  | 0.832 | -- |
| Lateral Ventricles, %change from baseline | |  |  |  |  |  |
|  | Number of subjects | 94 | 72 | 100 | 166 | 100 |
|  | Adjusted mean change (SE) | 8.76% (0.79) | 7.07% (0.89) | 10.74% (0.78) | 8.06% (0.67) | 10.76% (0.78) |
|  | Adjusted mean difference vs. placebo (95% CI) | -1.97% (-3.84 to -0.1) | -3.66% (-5.67 to -1.65) |  | -2.70% (-4.36 to -1.05) | -- |
|  | P value vs. placebo | 0.039* | <0.001*** |  | 0.002** | -- |

Volumetric MRI scans were performed at baseline and 48 weeks and used to calculate the annualized percent volume changes from baseline and difference of blarcamesine vs. placebo of defined brain regions and cavity for lateral ventricles, respectively. Listed regions include whole brain, total grey matter, total white matter, and lateral ventricles. Results are analyzed using a general linear model, with covariates of treatment group, baseline volume, and baseline MMSE grouping. Analysis was performed separately for individual treatment group comparisons and for combined active treatment group comparisons. Significance was evaluated using a threshold of 0.05. Asterisks [*, **, ***] indicate P < 0.05, 0.01, 0.001 respectively. Patient numbers in each treatment group represent patients with both baseline and end of study results for each measurement.

| **Supplemental Table 5. Primary and Secondary End Points, SIGMAR1 rs1800866 Variant Non-Carrier Subgroup** | | | | | |
| --- | --- | --- | --- | --- | --- |
|  | **Individual Group Comparison** | | | **Group Comparison** | |
|  | **Blarcamesine**  **30 mg**  **(N = 106)** | **Blarcamesine**  **50 mg**  **(N = 93)** | **Placebo**  **(N = 101)** | **Blarcamesine**  **(N = 199)** | **Placebo**  **(N = 101)** |
| **Primary efficacy endpoints** | | | | | |
| Change from baseline to week 48 in the **ADAS-Cog13** score | | | | | |
| No. of participants at week 48 | 81 | 56 | 81 | 137 | 81 |
| Adjusted mean change | 2.384 | 2.329 | 4.686 | 2.340 | 4.657 |
| Adjusted mean difference vs. placebo (95% CI) | -2.302  (-4.398 to -0.206) | -2.357  (-4.656 to -0.058) | .. | -2.317  (-4.182 to -0.453) | .. |
| P value vs. placebo | 0.031* | 0.045* | .. | 0.015* | .. |
| Less decline, % | 49.1% | 50.3% | .. | 49.8% | .. |
| Change from baseline to week 48 in the **ADCS-ADL** score | | | | | |
| No. of participants at week 48 | 81 | 58 | 85 | 139 | 85 |
| Adjusted mean change | -5.865 | -6.506 | -6.867 | -6.171 | -6.898 |
| Adjusted mean difference vs. placebo (95% CI) | 1.002  (-1.205 to 3.209) | 0.361  (-2.058 to 2.779) | .. | 0.727  (-1.230 to 2.683) | .. |
| P value vs. placebo | 0.373 | 0.770 | .. | 0.466 | .. |
| Less decline, % | 14.6% | 5.3% | .. | 10.5% | .. |
| **Secondary efficacy endpoint**  Change from baseline to week 48 in the **CDR-SB** score | | | | | |
| No. of participants at week 48 | 80 | 57 | 85 | 137 | 85 |
| Adjusted mean change | 1.150 | 1.216 | 1.782 | 1.184 | 1.785 |
| Adjusted mean difference vs. placebo (95% CI) | -0.632  (-1.160 to -0.104) | -0.565  (-1.145 to 0.014) | .. | -0.601  (-1.070 to -0.133) | .. |
| P value vs. placebo | 0.019* | 0.056 | .. | 0.012* | .. |
| Less decline, % | 35.5% | 31.8% | .. | 33.7% | .. |
| **Exploratory endpoint**  Improvement from baseline to week 48 in the **CGI-I** score | | | | | |
| No. of participants at week 48 | 79 | 57 | 84 | 136 | 84 |
| Adjusted improvement | 4.544 | 4.502 | 4.782 | 4.527 | 4.783 |
| Adjusted mean difference vs. placebo (95% CI) | -0.238  (-0.495 to 0.018) | -0.280  (-0.560 to 0.000) | .. | -0.256  (-0.483 to -0.030) | .. |
| P value vs. placebo | 0.069 | 0.0497* | .. | 0.027* | .. |
| Less decline, % | 5.0% | 5.9% | .. | 5.4% | .. |

| **Supplemental Table 6. Primary and Secondary End Points, SIGMAR1 rs1800866 Variant Carrier Subgroup** | | | | | |
| --- | --- | --- | --- | --- | --- |
|  | **Individual Group Comparison** | | | **Group Comparison** | |
|  | **Blarcamesine**  **30 mg**  **(N = 45)** | **Blarcamesine**  **50 mg**  **(N = 42)** | **Placebo**  **(N = 58)** | **Blarcamesine**  **(N = 87)** | **Placebo**  **(N = 58)** |
| **Primary efficacy endpoints** | | | | | |
| Change from baseline to week 48 in the **ADAS-Cog13** score | | | | | |
| No. of participants at week 48 | 27 | 27 | 41 | 54 | 41 |
| Adjusted mean change | 4.759 | 4.727 | 6.326 | 4.714 | 6.306 |
| Adjusted mean difference vs. placebo (95% CI) | -1.567  (-4.644 to 1.509) | -1.599  (-4.686 to 1.488) | .. | -1.593  (-4.174 to 0.989) | .. |
| P value vs. placebo | 0.317 | 0.309 | .. | 0.225 | .. |
| Less decline, % | 24.8% | 25.3% | .. | 25.2% | .. |
| Change from baseline to week 48 in the **ADCS-ADL** score | | | | | |
| No. of participants at week 48 | 28 | 27 | 41 | 55 | 41 |
| Adjusted mean change | -8.624 | -8.398 | -9.335 | -8.460 | -9.275 |
| Adjusted mean difference vs. placebo (95% CI) | 0.711  (-3.061 to 4.483) | 0.937  (-2.842 to 4.716) | .. | 0.815  (-2.328 to 3.958) | .. |
| P value vs. placebo | 0.711 | 0.626 | .. | 0.610 | .. |
| Less decline, % | 7.6% | 10.0% | .. | 8.8% | .. |
| **Secondary efficacy endpoint**  Change from baseline to week 48 in the **CDR-SB** score | | | | | |
| No. of participants at week 48 | 27 | 27 | 41 | 54 | 41 |
| Adjusted mean change | 1.445 | 1.506 | 1.707 | 1.461 | 1.691 |
| Adjusted mean difference vs. placebo (95% CI) | -0.263  (-0.980 to 0.454) | -0.201  (-0.912 to 0.510) | .. | -0.230  (-0.826 to 0.367) | .. |
| P value vs. placebo | 0.471 | 0.578 | .. | 0.449 | .. |
| Less decline, % | 15.3% | 11.8% | .. | 13.6% | .. |
| **Exploratory endpoint**  Improvement from baseline to week 48 in the **CGI-I** score | | | | | |
| No. of participants at week 48 | 28 | 26 | 41 | 54 | 41 |
| Adjusted improvement | 4.756 | 4.666 | 5.039 | 4.724 | 5.047 |
| Adjusted mean difference vs. placebo (95% CI) | -0.283  (-0.696 to 0.130) | -0.373  (-0.794 to 0.048) | .. | -0.323  (-0.673 to 0.027) | .. |
| P value vs. placebo | 0.179 | 0.082 | .. | 0.070 | .. |
| Less decline, % | 5.6% | 7.4% | .. | 6.4% | .. |

Supplemental Table 7. Sensitivity Analysis - Tipping Point Analysis

|  | ADAS-Cog13 | | | |
| --- | --- | --- | --- | --- |
|  | Active Worsening | | Placebo Improving | |
| MAR | Placebo | Active | Placebo | Active |
| LS Mean (SE) | 5.664 (0.877) | 3.691 (0.805) | 5.664 (0.877) | 3.691 (0.805) |
| LS Mean Diff (SE) |  | -1.973 (0.788) |  | -1.973 (0.788) |
| p-value |  | 0.0124 |  | 0.0124 |
|  |  |  |  |  |
| Tipping Point 1 |  |  |  |  |
| Shift |  | 1.88 | -3.20 |  |
| Shifted Mean | 5.800 | 4.247 | 5.158 | 3.604 |
| Shifted Diff |  | -1.553 |  | -1.554 |
| p-value |  | 0.0499 |  | 0.0494 |
|  |  |  |  |  |
| Tipping Point 2 |  |  |  |  |
| Shift |  | 1.90 | -3.30 |  |
| Shifted Mean | 5.802 | 4.253 | 5.143 | 3.601 |
| Shifted Diff |  | -1.548 |  | -1.541 |
| p-value |  | 0.0506 |  | 0.0514 |

Results of the tipping point analysis compare the primary MMRM result, which uses the missing at random (MAR) assumption for all missing data, to an imputation model where data are missing not at random (MNAR), to find the point at which worsening of the active group or improvement of the placebo group would make the result non-significant (p > 0.05).
